# Activation of Toll-like receptor-2 by tumor associated matrix metalloproteinase-2 modulates dendritic cell function

**DOI:** 10.1186/2051-1426-2-S3-O21

**Published:** 2014-11-06

**Authors:** Emmanuelle Godefroy, Nina Bhardwaj

**Affiliations:** 1 New York Blood Center LFKRI, New York, NY, USA; 2 Icahn School of Medicine at Mt Sinai, NY, NY, USA

## 

Matrix metalloproteinases (MMPs) are zinc-dependent endopeptidases which degrade extracellular matrix proteins and modulate cell proliferation, migration, differentiation and angiogenesis. MMP-2, a member of the gelatinase subfamily of MMPs, participates in the remodeling and resolution of tissue injury and tumorigenesis. We recently identified an unexpected new role for MMP-2 in the modulation of innate immune function and in the differentiation of inflammatory T_H_2 responses in the tumor microenvironment [1]. Pre-exposure to MMP-2 inhibits IL-12 function and up-regulates OX40L expression by human dendritic cells (DCs). Enzymatically active MMP-2 causes degradation of the IFNAR1 chain of the type-I IFN receptor, reducing the ability of IFN beta to enhance transcription of the IL-12p35 subunit through STAT1 phosphorylation. In the absence of IL-12, OX40L now functions as a key co-stimulatory molecule for the priming of T_H_2 cells. Indeed, we have identified T_H_2 cells within the TIL compartment of melanoma specimens that produce IL-4, TNF and IL-13. However, the T_H_2 mechanism by which MMP-2 up-regulates OX40L is not known and the role of MMP-2-driven T_H_2 cells *in vivo *has not been determined. In this study, we specifically investigated how MMP-2 up-regulates OX40L on DCs to drive type-2 polarization and the physiologic role of MMP-2 imprinted DC in driving T_H_2 cells. We identified a novel physiological receptor, namely TLR2, for MMP-2 on DCs that, upon activation, up regulates OX40L and induces the production of TNF and IL-6. Significantly, MMP-2 acted as an adjuvant to prime TH2 cells in vivo towards protein antigens. Therefore, extracellular MMP-2 including that derived from tumors has the potential to locally affect DCs leading to modulation of immune responses in malignant diseases.
